# Opinions of European Dietitians About Communications Promoting Healthy Eating: An International Comparative Study

**DOI:** 10.3390/nu18172932

**Published:** 2026-09-07

**Authors:** Paweł Bryła, Beata Ciabiada-Bryła

**Affiliations:** 1 Department of International Marketing and Retailing, University of Lodz, 90-131 Łódź, Poland; 2 Department of Preventive Medicine, Medical University of Lodz, 90-752 Łódź, Poland; beata.ciabiada-bryla@umed.lodz.pl

**Keywords:** dietitians, nutrition communication, healthy eating promotion, social media, cross-national comparison

## Abstract

**Background/Objectives**: Dietitians play a pivotal role in promoting healthy eating, yet their perspectives on effective communication channels and campaign priorities remain under-researched, particularly in cross-national contexts. This study compared European dietitians’ opinions on communication channels and priority topics for healthy eating campaigns targeting different age groups. **Methods**: A cross-sectional survey was conducted among 131 dietitians from Poland (*n* = 56), France (*n* = 36), the United Kingdom (*n* = 26), and Germany (*n* = 13). Participants rated the importance of 19 communication channels on a 5-point scale and selected three priority topics from 16 options for children, adults, and elderly populations. Data were analysed using one-way ANOVA with Bonferroni post-hoc tests and Chi-square tests (significance at *p* < 0.05). **Results**: Interpersonal channels—dietitians’ own recommendations (M = 4.10) and recommendations from family/friends (M = 4.02)—were rated the highest across all countries. Significant national differences emerged for digital channels: Polish dietitians rated social media the highest, while British dietitians were the most sceptical (apps: Poland 3.73 vs. UK 2.81, *p* < 0.001). Limiting processed foods (60.3%) and increasing fruit/vegetable consumption (59.5%) were top child priorities, with national variations for fast food (Poland 28.6% vs. France 5.6%, *p* = 0.006). For elderly populations, dietary diversity (49.6%) and fruit/vegetable intake (42.7%) led in terms of priorities; salt limitation was prioritised only in Poland (23.2%) and France (16.7%), *p* = 0.017. **Conclusions**: While European dietitians agree on interpersonal communication and core nutritional messages, national contexts shape digital tool perceptions and campaign priorities. Culturally sensitive, targeted nutrition communication strategies are needed to address diverse population needs across Europe.

## 1. Introduction

In an era characterized by information abundance and the rapid proliferation of digital media, the communication of evidence-based nutritional guidance has become increasingly complex. The field of public health nutrition faces the dual challenge of countering widespread misinformation while effectively promoting healthy eating behaviours across diverse populations. Dietitians, as qualified health professionals with expertise in food and nutrition, occupy a pivotal position in this communication landscape. They serve not only as direct sources of dietary advice for individuals but also as trusted public voices capable of shaping population-level nutritional attitudes and behaviours [1].

The channels through which dietary advice can be disseminated have expanded dramatically in recent decades. Traditional mass media, including television, radio, and print publications, have been supplemented—and in some cases supplanted—by an array of digital platforms. Social media networks, mobile health applications, blogs, and professional websites now offer unprecedented opportunities for reaching diverse audiences with nutritional messaging [2]. Concurrently, the commercial food environment continues to communicate its own messages through product packaging, advertising leaflets, and in-store promotions, often competing directly with public health objectives [3].

Within this multifaceted communication environment, the perspectives of dietitians themselves remain surprisingly under-researched. While considerable attention has been devoted to understanding how consumers perceive nutritional information and which communication channels influence their dietary choices, far less is known about how nutrition professionals evaluate the relative importance of different communication vehicles. Dietitians occupy a unique vantage point: they possess expert knowledge of nutritional science, maintain direct contact with individuals seeking dietary guidance, and observe at close range which messages and media resonate with their clients. Their collective opinions therefore constitute a valuable source of insight for informing the development of effective public health communication strategies. Dietitians possess specialized knowledge in nutrition and dietary practices, making them well-equipped to evaluate the effectiveness and accuracy of health messages. Their insights ensure that campaigns are based on sound nutritional science and can effectively guide the public towards healthier eating habits [4,5]. Dietitians can help tailor health messages to different demographic groups, ensuring that the information is relevant and accessible. This is important because dietary needs and barriers can vary significantly across different populations. For example, young adults may be more motivated by messages focusing on appearance rather than health [6]. Dietitians can identify and address common barriers to healthy eating, such as lack of knowledge, time constraints, and cost concerns. By understanding these barriers, they can help design campaigns that offer practical solutions and support behavior change. A study highlighted the importance of addressing misconceptions and increasing self-efficacy regarding healthy food choices [7]. Dietitians can collaborate with other health professionals to integrate comprehensive health messages that combine nutrition with other aspects of healthy living, such as physical activity. This holistic approach can enhance the overall effectiveness of health promotion efforts [8]. Dietitians’ feedback on existing campaigns can help refine and improve future initiatives. Their professional experience and direct interaction with clients provide valuable insights into what works and what doesn’t in promoting healthy eating [9,10].

Furthermore, the professional practice of dietetics does not occur within a cultural vacuum. National contexts shape both dietary patterns and communication cultures in ways that may influence how dietitians perceive the utility of various channels and the priority they assign to different nutritional messages. Cross-national comparative research offers the opportunity to distinguish between universal principles of effective nutrition communication and those that are contingent upon specific cultural, economic, or healthcare system contexts [11]. Europe, with its diversity of culinary traditions, healthcare arrangements, and media landscapes, provides a particularly rich setting for such comparative inquiry. Yet to date, no published study has systematically compared the opinions of dietitians across major European countries regarding the communication channels and campaign topics they consider the most important for promoting healthy eating.

This study addresses this gap by presenting findings from an international comparative survey of dietitians and nutritionists from four major European countries: Poland, France, the United Kingdom, and Germany. These nations were selected to represent distinct geographical regions, healthcare system models, and food cultures within Europe. The study had three primary objectives: first, to identify which communication channels dietitians perceive as the most important for effectively promoting healthy eating and to examine whether these perceptions vary significantly across countries; second, to determine the topics that dietitians prioritize for healthy eating campaigns targeting three distinct age groups (children and young people, adults, and elderly individuals) and to explore cross-national differences in these priorities; and third, to investigate dietitians’ engagement with digital tools, including their knowledge of nutrition applications and their use of social media platforms for professional purposes.

By capturing the perspectives of practitioners who work at the interface between nutritional science and the public, this research aims to provide evidence-based guidance for the design of culturally sensitive nutrition communication strategies. Understanding how dietitians across Europe evaluate the communication landscape is essential for public health authorities, professional bodies, and health educators seeking to optimize the reach and impact of their messages. Moreover, as digital technologies continue to transform health communication, documenting current patterns of professional engagement with these tools establishes a baseline against which future developments can be measured. The findings presented here contribute to the growing body of literature on nutrition communication while offering practical insights for those engaged in the vital work of promoting healthy eating across the lifespan.

Therefore, the specific objectives of this study are: (1) to compare European dietitians’ perceptions of the importance of various communication channels for promoting healthy eating; (2) to identify their priority topics for campaigns targeting children, adults, and elderly populations; and (3) to examine cross-national differences in these perceptions and priorities while also exploring dietitians’ engagement with digital tools and nutrition apps.

## 2. Materials and Methods

The objective of this study was to gather the perspectives of dietitians from four major European countries: Poland, France, the United Kingdom, and Germany. Nutritionists were also permitted to participate. The questionnaire was designed by the first author of this submission and consulted with the Head of Department of International Marketing and Retailing, University of Lodz. It is partly based on previously used questionnaire in own research about health claims and nutrition claims [12]. The original version was in Polish. It was translated into English, French, and German with the use of specialised software, and the translations were checked by Polish academics who are experts in this thematic area and, at the same time, are fluent in the given foreign language. This survey was designed in such a way that it should allow us to fine-tune a questionnaire addressed to a large sample of consumers, which was our next step in the Opus research project. Therefore, apart from the proposed items, it contained many open-ended questions, providing the opportunity for the dietitians to supplement the catalogue of answers, used in the previous close-ended question. For instance, question number 1 was as follows: “1. In your opinion, what is the importance of the following instruments for the effective promotion of healthy eating? (Please mark each line)”. A list of answers was provided, and the next question was “2. If, in your opinion, there are other important instruments for promoting healthy eating, please indicate them: ….”. This kind of approach allowed us to check whether the proposed catalogues of answers were appropriate or whether some modifications were needed.

In Poland, the survey was distributed to all enterprises categorized under “dietetics” in the Polish Telephone Directories (https://www.pkt.pl/szukaj/dietetyka?selectedCategoryRefinement=Dietetyka, accessed on 31 July 2024), as well as through numerous Facebook groups for dietitians and select LinkedIn groups. Furthermore, an invitation to participate was posted on the https://dietetycy.org.pl/, accessed on 13 January 2025, portal, and an email request was sent to the Polish Society of Dietetics. In France, a database containing contact information for dietitians was acquired from Base-Emails.com. In the United Kingdom, contact details were obtained using the directories available at https://www.bda.uk.com/find-a-dietitian.html, accessed on 1 September 2025, https://www.nutritionist-resource.org.uk/, accessed on 1 September 2025, and https://practitioner-search.bant.org.uk/, accessed on 1 September 2025. In Germany, email addresses of dietitians were sourced from https://www.vdd.de/diaetassistenten-suche/, accessed on 31 July 2024. In total, 131 completed questionnaires were received, comprising 56 from Poland, 36 from France, 26 from the United Kingdom, and 13 from Germany. The underlying causes of the small sample sizes are low response rates and abandoning the survey before its completion. In Germany, we addressed the invitation to complete the questionnaire to 919 dietitians—all those whose emails were available at https://www.vdd.de/diaetassistenten-suche/, accessed on 31 July 2024. We did not identify any other public directory of German dietitians or nutritionists. We received only 31 answers from Germany. As 18 answers were incomplete, we qualified 13 for further analyses. In the UK, we addressed the invitations to complete the questionnaire in 3 online channels allowing us to send direct messages to dietitians and nutritionists: 373 in https://www.bda.uk.com/find-a-dietitian.html, accessed on 1 September 2025, 637 in https://www.nutritionist-resource.org.uk, accessed on 1 September 2025, and 2407 in https://practitioner-search.bant.org.uk/, accessed on 1 September 2025. Overall, we received 112 responses, out of which 86 were incomplete. Therefore, we qualified 26 for further analyses. The issue of missing data in the surveys was also apparent in France and Poland. In France, we purchased a database of e-mails of dietitians with 5390 email addresses. We received 133 questionnaires, out of which 97 were incomplete. In Poland, we identified 1117 entities in the PKT database in the category of dietetics, and we extracted 703 emails. Additionally, we used a variety of contact possibilities in social media, as mentioned above. We received 182 responses, out of which 126 were not usable. The datasets generated and analysed during the current study are available in the Zenodo repository [13].

Data analysis was conducted using IBM SPSS 29 software. For nominal variables, cross-tabulation and Chi-square tests were employed. Mean comparisons across countries were performed using one-way analysis of variance (ANOVA), with post-hoc tests utilizing Bonferroni correction to identify specific inter-country differences. The ANOVAs concerned the opinions about the importance of selected communication channels for the effective promotion of healthy eating (which were coded in 1–5 scales). To account for the multiplicity of statistical tests resulting from the comparison of 19 communication channels across four countries, we controlled the false discovery rate using the Benjamini–Hochberg procedure with a 5% significance level. This approach was chosen to balance the risk of Type I errors with the need to identify meaningful cross-national differences in this exploratory comparative study. For the post-hoc pairwise comparisons following each ANOVA, we applied a stricter Bonferroni correction to control for within-family error rates. Open-ended questions were analysed by identifying the most recurrent themes.

The proportion of female participants in the sample was notably high, at 90.8 percent, with minor variation across countries: Poland, 87.5%; France, 88.9%; the United Kingdom, 100.0%; and Germany, 92.3%. These differences in gender distribution were not statistically significant (χ^2^(3) = 3.571, *p* = 0.312).

The mean age of respondents was 42.6 years, with a slightly lower median of 41 years. Age varied significantly across countries; Polish respondents were the youngest (M = 35.9, SD = 8.5), while their British counterparts were the oldest (M = 53.0, SD = 10.4). German respondents were also in their early 50 s (M = 50.8, SD = 9.3), while French ones were close to the international sample average age (M = 42.7, SD = 10.8). Post-hoc tests with Bonferroni correction revealed significant differences between Poland and France (*p* = 0.006), Poland and the United Kingdom (*p* < 0.001), Poland and Germany (*p* < 0.001), and France and the United Kingdom (*p* < 0.001).

The surveyed dietitians resided in diverse environmental settings. Nearly one-third lived in large cities, while approximately one-fifth resided in rural areas. Differences in place of residence across countries were not statistically significant.

Over three-quarters of the dietitians surveyed reported operating their own business or company. This varied significantly across countries; all respondents from the United Kingdom were self-employed, whereas only half of Polish dietitians held this status. Approximately one-quarter of respondents were employed under contract. Again, substantial international differences were observed, with the German subsample containing the highest proportion of employees. It is noteworthy that some dietitians combined self-employment with paid employment, particularly in Western Europe. Conversely, a considerable proportion of Polish dietitians reported neither status, which may be attributable to their younger age and ongoing educational pursuits. A total of 72.5 percent of all respondents indicated they had a professional website, with the highest prevalence observed in the United Kingdom and France.

The sample exhibited considerable diversity in terms of professional experience as a dietitian or nutritionist. The mean duration of professional experience was 11.4 years overall; however, this figure was more than twice as high among German respondents (M = 23.9, SD = 11.2). The shortest professional experience was observed in Poland (M = 7.2, SD = 5.4). The data for France were M = 13.5 and SD = 10.9, and for the UK, M = 11.3 and SD = 8.4. Post-hoc tests with Bonferroni correction confirmed significant differences between Poland and France (*p* = 0.004), Poland and Germany (*p* < 0.001), France and Germany (*p* = 0.001), and the United Kingdom and Germany (*p* < 0.001).

The study employed a mixed-methods approach, combining quantitative closed-ended items with qualitative open-ended questions. This design was selected to capture both the breadth of dietitians’ opinions across countries (quantitative) and the depth of their reasoning and contextual insights (qualitative). The open-ended questions were designed to complement the forced-choice items, allowing participants to elaborate on topics of personal significance and to identify communication channels or campaign themes not anticipated in the survey development process.

All procedures performed in this study were conducted in accordance with the ethical standards of the University of Lodz. Informed consent was obtained from all participating individuals. The research was approved by the Research Ethics Committee of the University of Lodz (approval no. 142/KEBN-UŁ/2025-26 of 14 May 2026).

## 3. Results

Table 1 and Table A1 present the findings from a study comparing the opinions of dietitians regarding the importance of various communication channels for promoting healthy eating. The answers were measured with 5-point labelled scales (very big, rather big, medium, rather small, and meaningless) and coded as 5-1. One-way ANOVAs were conducted to test for statistically significant differences between the countries for each channel. To control the family-wise error rate across the 19 ANOVAs, the Benjamini–Hochberg false discovery rate procedure was applied. Channels with a Benjamini–Hochberg-adjusted *p* < 0.05 were considered significant. Post-hoc pairwise comparisons were conducted using the Bonferroni correction. Looking at the “Total” column, dietitians across all four countries rated Dietitian’s recommendations (4.10) and Recommendations from family or friends (4.02) as the most important channels. This suggests a strong consensus that interpersonal trust and expertise are paramount for effective promotion. Dietitian’s profile on social media (4.08) and Consumer opinions on social media (3.95) also received very high total scores, highlighting the growing influence of digital and peer-to-peer communication. Channels with the lowest total mean scores were Shop advertising leaflets (2.75), Manufacturer’s website (2.81), and In-store salesperson recommendations (2.89). This indicates that traditional, commercial, and less personalized marketing materials are perceived as the least effective by dietitians. The one-way ANOVAs revealed that for several channels, the differences in opinions between countries were statistically significant. The most pronounced differences were found for digital and social media channels, as well as professional endorsements. The Bonferroni post-hoc tests pinpoint exactly which countries differed.

Here is a breakdown of the channels where dietitians’ opinions varied significantly by country:

1. Social Media Channels

Consumer opinions on social media (F = 9.215, *p* < 0.001): Polish dietitians rated this as extremely important (4.38), while their counterparts in the UK (3.42) and France (3.64) gave it much lower scores. Germany (4.00) was in the middle. The differences between Poland and both the UK and France were statistically significant (both *p* < 0.001).

Retailer profile on social media (F = 5.132, *p* = 0.002): Again, Polish dietitians saw this as considerably more important (3.73) compared to French dietitians (2.83) (*p* < 0.001).

Dietitian’s profile on social media (F = 4.897, *p* = 0.003): While highly rated overall, Polish dietitians were the strongest proponents (4.41). French (3.86) and British (3.65) dietitians, while still rating it above the midpoint, were less enthusiastic. The significant differences between Poland and both France (*p* = 0.037) and the UK (*p* = 0.005) reinforce the pattern that Polish dietitians place a greater premium on social media as a professional tool.

2. Digital and Technology Channels

Nutrition apps for smartphones (F = 6.673, *p* < 0.001): There is a clear divide. Dietitians in Poland (3.73) and France (3.50) view apps as moderately important, whereas dietitians in the UK (2.81) and Germany (3.15) expressed more cautious opinions. The significant differences between Poland and the UK (*p* < 0.001), and between France and the UK (*p* = 0.021), show that British dietitians are particularly unpersuaded by the potential of nutrition apps compared to their continental European colleagues.

Cooking blogs (F = 4.491, *p* = 0.005): Polish dietitians found cooking blogs to be of above-average importance (3.48), while German dietitians rated them as the least important of all channels in the entire study (2.54). The significant difference between Poland and Germany (*p* = 0.010) highlights a stark contrast in the perceived value of this type of content.

3. Professional Endorsements

Dietitian’s website (F = 3.938, *p* = 0.010): Consistent with other digital trends, Polish dietitians saw their own professional websites as very important (4.09), while dietitians in the UK (3.35) were less convinced. The significant difference between Poland and the UK (*p* = 0.009) supports this pattern.

4. Traditional Marketing Channels

Product packaging (F = 3.493, *p* = 0.018): Polish dietitians rated this as quite important (3.86), whereas German dietitians gave it a much lower score (2.92), making it one of the lowest-rated channels for Germany. There was a significant difference between Poland and Germany (*p* = 0.012).

The respondents were provided with the possibility to indicate additional communication channels for the effective promotion of healthy eating that they considered as important. First and foremost, dietitians strongly emphasize the need for structured and accessible education. They argue that effective promotion requires moving beyond what one respondent described as “a few advertising slogans” because nutrition is far too complex to be conveyed that way. The most frequently mentioned setting for this is school-based education. Suggestions include teaching children directly, for instance through interactive computer games with reward mechanics for correct answers, as well as educating their parents. Specific institutions mentioned include primary schools, middle schools, high schools, and parent–teacher meetings. Extending this education into adult life, dietitians also suggested workplace information sessions, community-based initiatives, and collective workshops. Furthermore, one respondent explicitly mentioned the value of formal courses and training conducted in easily accessible local units.

Secondly, the dietitians highlighted the importance of the healthcare system and professional endorsements. While the quantitative data already covered dietitians’ own recommendations, these open-ended responses point to a broader network of trusted professionals and official bodies. Respondents pointed to the wider healthcare team, including doctors, general practitioners (GPs), school nurses, physiotherapists, and nutritional therapists. One respondent noted that while dietitians can be more prescriptive, nutritional therapists also have a role in supporting general health and well-being. There is also a clear demand for authoritative, factual sources to combat misinformation. Specific examples of trusted websites given included Poland’s NCEZ (National Centre for Health Education), France’s *ameli* (Health Insurance), *HAS* (High Authority of Health), and *ARS* (Regional Health Agencies). More generally, dietitians mentioned government websites promoting health. Additionally, one respondent mentioned the importance of popular science books as a channel for disseminating nutritional knowledge.

Thirdly, regarding influencers, social media, and modern platforms, the qualitative responses add significant nuance by specifying which types of online presence are important. Specific platforms such as TikTok and Instagram are explicitly named, indicating that dietitians recognize the need to be present on platforms popular with younger demographics. The responses also differentiate between various types of online personalities. They highlight the importance of professional content creators and the profiles of well-known people in social media. The concept of “influencers and their opinion” was simply stated. However, a French respondent provided a crucial warning about celebrities, mentioning “influencers, stars, all known personalities who talk about nutrition without knowing.” This serves as a reminder that celebrity endorsements are a double-edged sword; they are influential but often lack the necessary expertise.

Finally, the dietitians pointed to the need for contextual, environmental, and systemic approaches, suggesting that promotion should not happen in a vacuum but should be integrated into people’s surroundings and address broader issues. For instance, school cafeterias are mentioned as a practical, everyday setting where healthy eating can be promoted and modelled. One French respondent simply stated the need to “fight against marketing,” implying that health promotion must actively compete with or counteract commercial food marketing. A particularly sophisticated comment suggested “recontextualizing food within a global approach,” linking it to overall health and well-being rather than just isolated nutrients. Lastly, a German respondent mentioned the importance of practical considerations like food tolerance and availability, highlighting that promotion is ineffective if it ignores individual dietary needs or a person’s ability to access the recommended ingredients.

The underlying message from all these responses is clear: dietitians believe that promotion is the most effective when it is educational, authoritative, and embedded in daily life. They are calling for a shift from passive advertising towards active engagement through schools, workplaces, and trusted healthcare professionals while simultaneously acknowledging the power—and potential dangers—of modern platforms like TikTok and influencers. The ultimate goal is to equip people with the skills and knowledge they need to navigate a complex world of nutritional misinformation.

The respondents were asked to indicate the 3 most important topics of campaigns promoting healthy eating among children and young people from a list of 16 options, including the possibility to specify another topic (Table 2). Across all four countries, two topics stand out as the most frequently selected priorities. Limiting the consumption of highly processed foods is the top priority, with 60.3% of dietitians overall choosing it. Increasing the consumption of vegetables and fruit is a very close second, selected by 59.5% of respondents overall, with particularly strong support in the UK. The statistical tests reveal significant differences in opinion for four specific topics, indicating that national context influences what dietitians view as the most urgent. In terms of increasing fibre intake, there is a major divergence, as this is a high priority for dietitians in the UK, at 42.3%, and France, at 36.1%, but a very low priority for those in Poland, at 10.7%, and Germany, at 7.7%. Regarding limiting the consumption of fast food, this is a far more pressing issue for Polish dietitians, at 28.6%, compared to their colleagues in France, at 5.6%, and the UK, at 3.8%. In terms of limiting the consumption of high-calorie snacks, French dietitians are the most concerned, at 19.4%, while it is not a priority at all for dietitians in the UK, at 0.0%. Several other notable country-specific observations can be made. German dietitians stand out for placing a much higher priority on increasing dietary diversity, at 46.2%, and limiting sugar consumption, also at 46.2%, compared to the overall average. They are also the least concerned with increasing fruit and vegetable consumption. Polish dietitians, aside from their focus on fast food, show a concern for limiting energy drinks, at 21.4%, compared to other countries, while they show the lowest priority for increasing fibre intake. The UK has the strongest focus on the two most popular topics, with the highest priority for increasing fruit and vegetables, at 69.2%, and increasing fibre intake, at 42.3%, and they are also the most interested in promoting reading food labels, at 26.9%. French dietitians have the highest level of concern for limiting highly processed foods, at 69.4%, and they are also the most concerned about high-calorie snacks. Finally, several topics were consistently ranked as very low priorities across all countries. These include limiting salt intake, limiting saturated fats, and limiting trans fats, all of which received less than 5% of the vote overall. This suggests that dietitians view other dietary issues as more pressing for this demographic.

In addition to the pre-defined topics, the surveyed dietitians from the four European countries proposed several other important themes for campaigns promoting healthy eating among children and young people. These suggestions reflect a broader, more holistic approach to nutrition education. Dietitians from Poland emphasized the importance of properly composing meals with the right balance of proteins, fats, and carbohydrates. They illustrated this concept with a practical example, suggesting that an apple should not be presented as a full meal but rather as a component of one, recommending that children should add nuts or natural yogurt to it. French dietitians proposed two distinct topics. The first is education about taste, which involves developing children’s palates and appreciation for different flavours. The second, and perhaps more psychologically focused topic, is body dissatisfaction. They noted that this issue is known to cause eating disorders and other eating-related problems, suggesting that campaigns should address mental and emotional health alongside physical nutrition. Dietitians from the United Kingdom focused on the practical skill of cooking, proposing that campaigns should encourage preparing meals at home. This suggests a focus on reconnecting young people with whole foods and the process of meal preparation. Finally, German dietitians proposed a reduction in meat consumption, with a specific emphasis on limiting processed products and red meat. They also highlighted the critical role of formal education settings by recommending that nutritional education should be integrated into kindergarten and school curricula.

Table 3 presents the priorities of dietitians from the four European countries, regarding the topics that should be the focus of healthy eating campaigns for adults. The respondents were asked to select the three most important topics. Across all four countries, two topics emerge as the most frequently selected priorities for adults. Increasing the consumption of vegetables and fruit is the top priority, with 49.6% of dietitians overall choosing it. Limiting the consumption of highly processed foods is a very close second, selected by 47.3% of respondents overall. The statistical tests reveal significant differences in opinion for four specific topics, indicating that national context significantly influences what dietitians view as the most urgent for the adult population. For increasing fibre intake, there is a major divergence in priorities. This topic is a high priority for dietitians in the UK, at 50.0%, and France, at 41.7%, but a much lower priority for those in Germany, at 23.1%, and especially Poland, at only 14.3%. Regarding the limitation of high-calorie snacks, German dietitians stand out dramatically. More than a third of German respondents, 38.5%, selected this as a top priority, which is significantly higher than in any other country. In comparison, only 7.1% of Polish dietitians, 7.7% of British dietitians, and just 2.8% of French dietitians considered this a priority. In terms of limiting salt intake, Polish dietitians show a concern. While 14.3% of Polish respondents selected this topic, it was not considered a priority at all by dietitians in the UK or Germany, and only 2.8% of French dietitians selected it. Similarly, limiting the consumption of saturated fats was a concern almost exclusively for Polish dietitians. Over a tenth of Polish respondents, 10.7%, prioritized this topic, whereas it was not selected by a single dietitian in France, the UK, or Germany. Several other notable country-specific observations can be made. German dietitians placed the highest priority on increasing dietary diversity, at 61.5%, and limiting sugar consumption, at 46.2%, compared to their European colleagues. In contrast, British dietitians showed the strongest support for increasing fruit and vegetable consumption, at 65.4%. Polish dietitians were the most concerned about limiting alcohol consumption, with 37.5% selecting this as a top priority, which is notably higher than in the other countries. Finally, several topics were consistently ranked as very low priorities across all countries. These include increasing the consumption of milk and dairy products, limiting the consumption of energy drinks, and limiting the consumption of trans fats. This suggests that dietitians view other dietary issues as more pressing for the adult population. Only 2 respondents used the option to supplement the catalogue of answers with their own proposition, which suggests that our framework was appropriate. One French dietitian added cooking home with raw ingredients, and one German dietitian indicated meat consumption reduction.

Table 4 presents the findings of the survey conducted among dietitians regarding the topics they believe should be prioritized in healthy eating campaigns for elderly people. The respondents were asked to select the three most important topics, and the results are displayed as the percentage of dietitians in each country who chose that specific topic. Overall, the two most frequently mentioned priorities across all countries combined were “Increasing dietary diversity,” which was cited by nearly half of all respondents at 49.6 percent, and “Increasing the consumption of vegetables and fruit,” which was chosen by 42.7 percent of dietitians. In contrast, the topics considered the lowest priority were “Limiting the consumption of energy drinks” and “Limiting the consumption of fast food,” each receiving less than one percent of the votes across the entire sample. When examining the results by country, several distinct priorities and patterns emerge. For the top overall priority of increasing dietary diversity, German dietitians placed the greatest emphasis on this topic at 61.5 percent, followed closely by Poland at 55.4 percent. In comparison, dietitians in the United Kingdom ranked this as a lower priority, with only 34.6 percent selecting it. Regarding the consumption of fruits and vegetables, this was seen as the most critical in the United Kingdom, where 50 percent of dietitians chose it, and in Germany, with 46.2 percent. However, it was a notably lower priority in France, where only one-third of respondents, or 33.3 percent, selected this option. The statistical analysis, represented by the *p*-values, reveals where the differences between countries are the most significant and unlikely to be due to chance. The most statistically significant difference was found in the topic of “Limiting salt intake,” with a *p*-value of 0.017. This priority was mentioned exclusively by dietitians in Poland and France, at 23.2 percent and 16.7 percent respectively. Remarkably, no dietitians in both the United Kingdom and Germany considered limiting salt to be one of the three most important topics for the elderly. A similarly significant difference, with a *p*-value of 0.018, was observed for “Limiting the consumption of highly processed foods.” Dietitians in the United Kingdom ranked this as a much higher priority than their counterparts elsewhere, with 38.5 percent selecting it. In contrast, only 12.5 percent of Polish, 13.9 percent of French, and just 7.7 percent of German dietitians chose this topic. The data also shows near-significant differences that highlight interesting national trends. For “Increasing the consumption of milk and dairy products,” which had a *p*-value of 0.056, there is a clear divide. German and French dietitians prioritize this highly, at 46.2 percent and 38.9 percent respectively, likely due to concerns about bone health and osteoporosis in the aging population. However, this topic was a very low priority in the United Kingdom, at only 15.4 percent, and in Poland, at 21.4 percent. In summary, the results suggest that while European dietitians generally agree on the importance of promoting dietary diversity and increasing fruit and vegetable intake for the elderly, there are significant national differences in opinion regarding restrictive messages. The United Kingdom appears more focused on limiting processed foods, Poland places emphasis on reducing salt, and Germany prioritizes increasing consumption of various food groups over imposing dietary restrictions.

Based on the raw data provided in the “Other” category of the study, here is a summary of the additional campaign topics suggested by European dietitians when they were asked to list priorities not already covered in the main list. The overwhelming theme among the “write-in” responses was a focus on protein intake. Another significant topic that emerged was hydration. This highlights the importance of fluid intake as a critical, yet sometimes overlooked, aspect of elderly nutrition. German dietitians contributed a focus on the broader concept of prevention and adequacy, with suggestions like “Prävention von Mangelernährung” (prevention of malnutrition) and “Bedarfsgerechte Ernährung” (needs-based nutrition). A French dietitian also emphasized the psychological aspect of eating in old age by suggesting campaigns should “garder le plaisir de manger” (keep the pleasure of eating). Finally, a Polish respondent made a connection between nutrition and broader health by suggesting the topic of “Żywienie a choroby cywilizacyjne,” which translates to “Nutrition and diseases of civilization.”

The self-reported knowledge of any nutritional apps for smartphones that are useful in promoting healthy eating differed significantly across the country samples (Chi^2^(3) = 10.326, *p* = 0.016): Poland (69.6%), France (47.2%), UK (34.6%), Germany (61.5%). In total, 55.7% of the surveyed dietitians indicated they knew any nutrition apps.

Here is a summary of the nutrition apps spontaneously mentioned by European dietitians as useful tools for promoting healthy eating, organized by their primary function and country of mention. The most frequently mentioned app was Fitatu, a Polish-developed calorie counter and diet planner. It was cited dozens of times, often in combination with other apps, indicating it is a dominant tool in Poland. Other popular calorie-counting and tracking apps mentioned include MyFitnessPal, Yazio, and Lifesum, which were referenced by dietitians in the UK, Germany, and Poland. A significant category of apps focused on scanning food labels and assessing product quality. In Poland, the apps Zdrowe Zakupy (Healthy Shopping) and Czytaj Skład (Read the Ingredients) were frequently mentioned together. In France and the UK, dietitians recommended Yuka and Open Food Facts, which allow users to scan barcodes and receive a health rating for food products. The German mention of apps from consumer centres (“Verbraucherzentrale”) aligns with this interest in informed shopping. Several apps are designed for specific dietary management or medical conditions. The Freshwell app was mentioned by a UK dietitian for its low-carb approach aimed at helping people with type 2 diabetes. In Germany, apps associated with statutory health insurance companies, such as Ovida, were noted. These are often prescribed or recommended for conditions like obesity or diabetes and include professional coaching. Finally, there were mentions of apps focused on food waste and sustainability, such as the German initiative “Zu gut für die Tonne” (Too good for the bin), as well as broader health and recipe apps like Jow (a meal planning app) and ZOE (a personalized nutrition program).

The self-reported extent of using social media as a dietitian (or nutritionist) was close to the scale midpoint in all the four countries under study: Poland (2.89), France (2.50), UK (2.46), Germany (2.31). The mean for all answers was 2.64. The international differences were not significant (F = 1.561, *p* = 0.202).

Table 5 presents data on the percentage of dietitians who maintain a professional social media profile. The respondents were asked which platforms they use, and the results show significant variation in platform preference both overall and between nations. Across all four countries combined, Facebook is the most widely used social media platform among dietitians, with 61.1 percent of respondents maintaining a profile there. It is closely followed by Instagram, used by 55.0 percent of dietitians overall. Professional networking platforms also feature prominently, with LinkedIn being used by just over half of all respondents at 52.7 percent. In contrast, platforms such as Twitter (X) and TikTok have very low adoption rates among dietitians, at 4.6 percent and 12.2 percent respectively, while YouTube remains a niche platform at 10.7 percent. The Chi-squared tests reveal that the differences between countries are highly significant for two platforms in particular. The most striking variation is seen with LinkedIn, which has a *p*-value of less than 0.001, indicating a extremely significant difference. This platform is overwhelmingly popular among dietitians in the United Kingdom and France, with 76.9 percent and 69.4 percent respectively maintaining a profile. It is moderately used in Poland at 37.5 percent, but it is the least popular in Germany, where only 23.1 percent of dietitian respondents use it. A significant difference was also found for Twitter (X), with a *p*-value of 0.024. The platform is almost exclusively used by dietitians in the United Kingdom, where 15.4 percent have a profile. In contrast, usage is at zero percent in both France and Germany, and only 3.6 percent in Poland. Polish dietitians show the most diverse platform usage. They have the highest adoption of TikTok (19.6%) and are the only users of the platform besides the UK. They also maintain a strong presence on the leading platforms of Facebook (62.5%) and Instagram (57.1%). French dietitians are heavily focused on the top three platforms. They have the highest usage of Facebook (75.0%) among all countries, a very high usage of LinkedIn (69.4%), and a strong presence on Instagram (58.3%). They show zero interest in Twitter (X). UK dietitians are the clear leaders in professional networking and microblogging. They have the highest usage of LinkedIn (76.9%) and are the primary users of Twitter (X) (15.4%). Their Facebook usage (50.0%) is moderate compared to France and Poland. German dietitians are the least engaged with the major social media platforms overall. They have the lowest usage of Facebook (38.5%), Instagram (30.8%), and LinkedIn (23.1%). They report zero usage of Twitter (X) and TikTok. Their only platform usage that aligns with others is YouTube, at 7.7 percent.

## 4. Discussion

### 4.1. Interpretation of Results

A striking finding across all four countries was the consistent ranking of interpersonal communication channels as the most important for promoting healthy eating. Dietitians’ own recommendations, alongside those from family and friends, received the highest mean importance scores overall. This underscores the enduring value of trust-based, personalized advice in nutrition communication, a finding that aligns with established health communication theories emphasizing the role of source credibility and social influence in behaviour change [14]. The Health Belief Model and Social Cognitive Theory both posit that individuals are more likely to adopt health behaviours when messages come from credible sources and are reinforced within their social networks [15]. Despite the proliferation of digital media, dietitians continue to view the human element—whether professional or personal—as irreplaceable in conveying nutritional guidance. However, it is essential to recognize that these ratings reflect dietitians’ subjective professional judgements rather than objective evidence of behavioral effectiveness. While dietitians’ perceptions are valuable for understanding professional consensus and identifying potentially promising approaches, the fact that a channel is highly rated by practitioners does not necessarily mean it will produce measurable changes in eating behaviors when implemented in real-world settings. The distinction between professional opinion and evidence-based effectiveness is critical, as communication channels that appear intuitively credible to health professionals may not always translate into sustained dietary improvements among target populations.

While consensus existed on the value of interpersonal communication, significant cross-national differences emerged regarding digital and social media channels, while previous research indicated that the use of social media is a common strategy for encouraging healthier diets [16]. Polish dietitians consistently rated the importance of various social media platforms, including consumer opinions, retailer profiles, and dietitians’ own profiles, significantly higher than their counterparts in other countries. They also placed greater emphasis on nutrition apps, cooking blogs, and their own professional websites. The relatively younger age of the Polish subsample, with a mean age of 35.9 years compared to 53.0 years in the United Kingdom, likely contributes to this greater enthusiasm for digital tools. Younger professionals may be more immersed in digital culture and more optimistic about its potential for health promotion, a phenomenon observed in research on digital natives and technology adoption.

In stark contrast, British dietitians exhibited marked skepticism towards digital channels. They rated nutrition apps, social media platforms, and even their own professional websites and recommendations lower than their Polish colleagues. Several possible explanations for this pattern warrant consideration, though they remain speculative given the study’s design. One hypothesis is that the United Kingdom’s longer history of professional regulation and established practice patterns within the National Health Service may foster greater reliance on traditional clinical communication models [1]. However, it is important to note that this interpretation is inferential, as the study did not directly measure dietitians’ attitudes towards regulation or their perceptions of NHS practice norms. A more directly supported explanation relates to the demographic characteristics of the British subsample: British respondents were the oldest, with a mean age of 53.0 years and mean professional experience of 11.4 years, potentially representing a cohort less inclined to embrace digital tools. Research on technology adoption in healthcare professions has consistently identified age and years of experience as significant predictors of digital engagement [17]. Another possible interpretation is that the skepticism may reflect differing professional cultures regarding the appropriate boundaries of digital health communication. The finding that British dietitians rated their own professional recommendations lower than those in other countries is particularly intriguing and could suggest greater professional humility regarding the limits of dietary advice in the face of complex behavioral determinants [18].

French dietitians reported the highest Facebook usage (75.0%) and high LinkedIn usage (69.4%) among the four countries, alongside moderate Instagram adoption (58.3%) and minimal TikTok engagement (5.6%). While these descriptive patterns suggest a preference for established platforms, the data do not permit firm conclusions about deliberate professional strategy. Several explanations are possible—including age-related platform preferences, broader national social media adoption patterns, or cultural norms regarding professional boundaries [19]—but these remain speculative hypotheses requiring direct empirical testing, as the study did not assess participants’ motivations, professional norms, or cultural orientations.

German dietitians presented a distinctive pattern characterized by scepticism towards specific channels—notably cooking blogs and product packaging—while maintaining high regard for interpersonal recommendations. Their low engagement with social media platforms overall, with only 38.5% maintaining Facebook profiles and 30.8% using Instagram, suggests either a more reserved professional culture regarding online presence or different patterns of patient communication within the German healthcare system. The high proportion of German respondents who were employees may influence their perceived need for professional online visibility, as employed professionals may rely on institutional rather than personal platforms for client communication [20].

There are additional contextual factors that contribute to cross-national differences in social media adoption by dietitians. They include privacy protection regulations, professional association guidelines, and social media ecosystem characteristics. While GDPR provides a common EU framework, national enforcement cultures and data protection sensitivities vary considerably. Germany exhibits a particularly strong privacy orientation (Datenschutzkultur), with strict enforcement by state data protection commissioners. Patient data protection guidelines explicitly caution German healthcare professionals against sharing identifiable clinical information on social media. This regulatory climate may contribute to German dietitians’ more reserved social media engagement. The UK, while maintaining GDPR alignment post-Brexit, operates within a different regulatory culture shaped by the NHS framework and HCPC oversight. France maintains a strict regulatory framework for health-related digital communication [21].

The British Dietetic Association (BDA) has developed extensive social media guidance for members, including platform-specific resources such as a “Dietitians’ Guide to TikTok” [22] and professional standards for online conduct. This proactive approach reflects an institutional strategy to “amplify visibility and influence”. Poland’s professional code of ethics has evolved to address media activity and online marketing, though formal guidance appears less prescriptive than in the UK [21].

Platform preferences vary significantly across countries. While LinkedIn dominates professional networking in the UK and France, Germany retains Xing as a locally preferred platform [21]. LinkedIn usage among dietitians in our sample reflected this pattern (UK 76.9%, France 69.4%, Poland 37.5%, Germany 23.1%). Broader adoption rates also differ: Germany reports relatively low overall social media use among individuals (59%) compared to the EU average [21], which may influence professional uptake. These ecosystem differences affect both the perceived utility of specific platforms and the effort required to maintain a professional presence.

Across all countries, limiting highly processed foods (60.3%) and increasing fruit and vegetable consumption (59.5%) emerged as the top priorities for campaigns targeting children and young people. This consensus reflects widespread concern about the nutritional quality of modern diets and the early establishment of dietary habits that track into adulthood [23]. The high prioritization of limiting processed foods aligns with growing evidence linking ultra-processed food consumption to adverse health outcomes in paediatric populations [24,25]. Polish dietitians stood out for their concern about limiting fast food consumption (28.6%), a priority notably lower in France (5.6%) and the United Kingdom (3.8%). This may reflect Poland’s more recent exposure to rapid expansion of fast food outlets and associated dietary changes following economic transformation. Studies have documented the nutrition transition in Central and Eastern European countries following EU accession, with increased availability of fast food and corresponding changes in dietary patterns [26]. French dietitians’ concern about high-calorie snacks (19.4%) and their exclusive mention of body dissatisfaction as an additional topic suggest greater attention to the psychological dimensions of eating and the specific eating patterns prevalent in French youth, consistent with research on the dual burden of malnutrition and body image concerns in European adolescents [27].

For the adult population, increasing fruit and vegetable consumption (49.6%) and limiting highly processed foods (47.3%) remained top priorities, maintaining consistency with the child-focused campaigns. These priorities reflect the strong evidence base linking these dietary factors to cardiovascular health, cancer risk, and overall mortality [28,29].

For the elderly, increasing dietary diversity (49.6%) and fruit and vegetable consumption (42.7%) emerged as top priorities, reflecting recognition of the specific nutritional challenges facing older adults, including reduced appetite, chewing difficulties, and social isolation affecting food intake [30,31]. Dietary diversity has been identified as a key indicator of nutritional adequacy in older populations and is associated with reduced mortality [32]. The significant national differences revealed distinct approaches to elderly nutrition. The United Kingdom’s focus on limiting highly processed foods (38.5%) for the elderly, contrasting with lower prioritization elsewhere, may reflect particular concerns about convenience food consumption among older Britons. Research has documented the increasing use of ready meals and processed foods among older adults in the UK, particularly those living alone [33]. Poland’s emphasis on salt limitation (23.2%) and saturated fat restriction (21.4%) suggests a continuation of cardiovascular-focused messaging across the lifespan, consistent with the high cardiovascular disease burden in the Polish population.

The significant national variation in the dietitians’ self-reported knowledge of nutrition apps (Poland 69.6%, Germany 61.5%, France 47.2%, United Kingdom 34.6%), reveals substantial differences in digital tool awareness that may influence professional practice. The dominance of Fitatu in Poland, mentioned dozens of times, indicates successful market penetration of a locally developed app and suggests that dietitians are engaging with tools relevant to their national context. Research on nutrition app usage has demonstrated considerable variation across European countries, influenced by factors including smartphone penetration, health system characteristics, and cultural attitudes towards technology [34]. However, dietitians’ awareness of apps should not be conflated with evidence of their effectiveness in promoting sustained dietary change. While these tools may be useful for self-monitoring and providing information, intervention studies have shown that app-based dietary interventions often suffer from high attrition rates and limited long-term impact. The professional enthusiasm for such tools, particularly in Poland, may reflect optimism about technological solutions rather than proven effectiveness, highlighting the need for rigorous evaluation of digital tools before they are widely recommended in clinical practice.

### 4.2. Study Limitations

Several limitations should be acknowledged. First, the sample sizes, particularly for Germany (n = 13) and the United Kingdom (n = 26), are relatively small, limiting the statistical power to detect differences and the generalizability of findings within these countries. The German subsample, while providing valuable insights, should be interpreted with particular caution given its size. Future research with larger, more representative samples would strengthen the evidence base. The substantial imbalance in country sample sizes introduces notable analytic constraints. Primarily, statistical power is asymmetrically distributed, with the German and UK samples exhibiting limited capacity to detect moderate effect sizes, thereby increasing the risk of Type II errors for these specific subpopulations. As a result, the precision of estimates is considerably lower for the smaller groups. Furthermore, the unbalanced design complicates cross-country comparability, as standard ANOVA results may be disproportionately weighted by the Polish majority, and Bonferroni-corrected post-hoc tests lose sensitivity in pairwise comparisons involving smaller samples. Given that variances may differ substantially across countries, particularly regarding digital tool perceptions, the observed significant differences should be interpreted cautiously, as the combination of unequal sample sizes and heterogeneous variances may inflate the risk of Type I errors.

Second, the sampling methods differed across countries due to the availability of contact information and professional directories. While this pragmatic approach was necessary to reach dietitians in each national context, it may have introduced selection biases that could affect cross-national comparisons. The use of different recruitment strategies across countries is a common challenge in international comparative research.

Third, we did not obtain information if the respondent was a dietitian or a nutritionist, and consequently, we could not compare their responses. As we do not have information about the share of nutritionists in each country sample, this mixed participant group may be a source of bias in cross-national comparisons if the proportions differ. We also acknowledge various academic backgrounds and professional statuses of dietitians and nutritionists in the countries under study.

Fourth, the study relied on self-reported opinions and practices rather than observed behaviour. Dietitians’ stated beliefs about the importance of various communication channels may not perfectly align with their actual practices or effectiveness. Social desirability bias may have influenced responses, particularly regarding professional behaviours and priorities.

Fifth, the cross-sectional design captures opinions at a single point in time, whereas both digital landscapes and professional attitudes evolve rapidly. The findings represent a snapshot that may not reflect current trends, particularly given the fast-changing nature of social media platforms. Longitudinal research would be valuable to track changes in professional attitudes over time.

Sixth, the study focused on dietitians’ perspectives rather than the views of the target populations (children, adults, and elderly individuals), who might have different opinions about which communication channels and messages are the most effective or appealing. Patient and public perspectives are essential for developing effective health communication strategies.

Seventh, the study’s interpretations of cross-national differences in digital tool perceptions, particularly those attributing skepticism to professional regulation, healthcare system characteristics, or professional culture, are speculative in nature. While demographic variables such as age and professional experience were directly measured, the study did not assess participants’ attitudes towards regulation, their perceptions of healthcare system norms, or their cultural orientations. Consequently, interpretations invoking these factors should be treated as hypotheses requiring direct empirical testing in future research rather than confirmed causal explanations.

A further limitation concerns the analysis of open-ended responses. The brief nature of qualitative responses, typical of survey-based data collection, limits the depth of insight achievable compared to in-depth interviews or focus groups. While thematic saturation appeared to be achieved, the absence of member checking (i.e., returning to participants to verify interpretations) may have introduced researcher bias in theme identification. Future studies would benefit from qualitative follow-up interviews to explore the themes identified here in greater depth.

Finally, the study did not examine the actual effectiveness of different communication channels or campaign topics. While dietitians’ professional opinions are valuable, they do not necessarily predict which approaches will be the most successful in changing eating behaviours. Experimental or intervention studies would be needed to establish effectiveness.

### 4.3. Implications for Practice and Policy

Despite these limitations, the findings offer several implications for practice and policy. The consistent prioritization of interpersonal communication channels suggests that investment in training dietitians in effective communication skills remains essential. While digital tools offer valuable supplements, they cannot replace the trusted relationship between professional and client. Communication skills training should remain a core component of dietetic education and continuing professional development.

We suggest the following country-specific communication strategies. In Poland, for children and adults, launch co-created campaigns with popular Polish food vloggers (e.g., ZdroweGotowanie) to promote “fast food swaps” and use TikTok/Instagram short-form videos showing local fast-food alternatives (e.g., zapiekanka vs. wholegrain toast), linked to the dominant Fitatu app for meal tracking. For the elderly, deploy printed materials with large fonts in pharmacies and GP clinics, focusing on salt reduction and protein/hydration (from open-ended themes). Use simple infographics showing practical swaps (e.g., replacing salt with herbs in traditional Polish soups). In France, for children, address the uniquely high concern for high-calorie snacks and body dissatisfaction via private Facebook groups for parents coordinated by school canteen staff. Integrate the Yuka app into school workshops to gamify snack label reading. For adults, leverage official government platforms to host “cooking with raw ingredients” video series, endorsed by dietitians on LinkedIn. Use supermarket loyalty app notifications (e.g., Carrefour) to nudge seasonal fruit/veg purchases. For the elderly, develop “Plaisir de Manger” recipe booklets adapted for swallowing difficulties, distributed via pharmacies, explicitly prioritizing dietary diversity and protein intake over restrictive messages. In the United Kingdom, for children, partner with the NHS Healthy Start scheme to integrate the Freshwell app (low-carb, diabetes-focused) into school cooking classes, addressing the highest priority for fiber and fruits/veg. For adults, use supermarket loyalty card data (Tesco, Sainsbury’s) to trigger in-app nudges for lower-processed ready-meal alternatives, aligning with the BDA’s professional guidelines. For the elderly, work with meal delivery services (e.g., Wiltshire Farm Foods) to offer dietitian-approved, lower-sodium versions of classic British meals, addressing the UK’s uniquely high focus on limiting processed foods for this age group. In Germany, for children and adults, avoid public social media campaigns. Instead, invest in official school and kindergarten curricula and workplace canteen interventions. Leverage the Verbraucherzentrale (consumer centers) for neutral, offline workshops on reducing high-calorie snacks. For the elderly, exclusively use positive, non-restrictive messaging via statutory health insurance patient portals (e.g., Ovida). Focus solely on needs-based nutrition and preventing malnutrition, as German dietitians showed zero prioritization of salt or processed food restriction for the elderly.

Regarding divergent app attitudes among the study participants, we provide the following country-specific recommendations. In the UK, overcome practitioner hesitation by establishing an official BDA app accreditation framework. Create a curated NHS-Approved or BDA-Validated list of evidence-based apps (e.g., Freshwell, MyFitnessPal). This reduces liability concerns and provides older/employed dietitians a safe, regulatory-backed pathway to recommend digital tools without needing deep personal tech engagement. In Poland, formalize Continuous Professional Development (CPD) points for completing accredited training on commercially dominant apps (Fitatu). Since many are self-employed, provide workshops on ethical app integration—teaching how to use app-generated data to enhance consultations rather than replace professional judgment while managing unrealistic client expectations. In Germany, leverage the German system of Digital Health Applications (DiGA). Advocate for specific nutrition apps to be listed on the DiGA directory, allowing physicians/dietitians to prescribe them via statutory insurance. This directly addresses privacy concerns by embedding apps within the trusted, regulated healthcare framework, moving from social media risk to prescribed therapy. In France, develop a standardized “App Interpretation Script” for dietitians to use during consultations. Since France has high app awareness for label scanners (Yuka, Open Food Facts), but moderate usage, provide training on explaining the limitations of single-score systems (e.g., nutrient-centric vs. holistic). Disseminate these scripts via the strong LinkedIn network to ensure uniform, evidence-based professional guidance.

Crucially, the findings should be interpreted as reflecting professional consensus on message content and preferred communication channels, not as evidence of the effectiveness of specific interventions. Public health authorities and campaign developers should use these insights as a starting point for developing interventions but should subsequently subject them to rigorous evaluation to determine whether the approaches favored by dietitians actually produce meaningful behavioral change. The distinction between professional endorsement and evidence-based practice must be maintained throughout the intervention development process, with pilot testing and outcome measurement serving as essential complements to expert opinion.

The substantial variation in digital tool perceptions across countries underscores the need for context-sensitive implementation of digital interventions. Rather than assuming that what works in one national context will transfer successfully to another, practitioners should consider conducting formative research and pilot studies to assess the acceptability, feasibility, and effectiveness of digital tools in their specific settings. The skepticism expressed by British dietitians, for example, may warrant particular attention to the evidence base for digital interventions before widespread adoption is recommended.

Finally, the pattern of social media adoption suggests that professional bodies and nutrition organizations should consider platform-specific strategies for engaging with dietitians. LinkedIn’s strong adoption in the United Kingdom and France makes it a suitable channel for professional development content in those countries, while Facebook’s broader reach across all countries suggests it remains a reliable platform for general professional communication. The emerging use of TikTok among younger Polish dietitians may signal future trends that could spread to other countries as digital natives comprise larger proportions of the profession, suggesting that professional bodies should monitor platform adoption patterns and adapt their communication strategies accordingly.

## 5. Conclusions

This international comparative study reveals that European dietitians share fundamental agreement on the importance of interpersonal, trust-based communication and core nutritional priorities such as increasing fruit and vegetable consumption and limiting highly processed foods. At the same time, significant national differences were observed in dietitians’ perceptions of digital channels and in the specific topics they prioritized for campaigns targeting different age groups.

The observed patterns suggest several descriptive trends: Polish dietitians rated digital tools more highly than their counterparts in other countries; British dietitians expressed greater skepticism towards digital channels while showing strong consensus on core nutritional messages; French dietitians demonstrated high engagement with established platforms like Facebook and LinkedIn; and German dietitians prioritized positive messages of increasing consumption over restrictive messages and showed more reserved engagement with social media.

These cross-national differences may be attributable to various factors, including demographic characteristics such as age and professional experience, as well as differences in practice contexts, healthcare system structures, and cultural orientations towards food and health. However, the present study did not directly assess these underlying mechanisms, and such explanations should therefore be treated as hypotheses for future investigation rather than confirmed causal relationships. Research employing qualitative methods or specifically designed quantitative measures would be valuable to test these potential explanations and clarify the drivers of cross-national variation in dietitians’ communication preferences.

The findings underscore the importance of culturally sensitive approaches to nutrition communication and the need for continued attention to the evolving digital landscape in which dietitians practice. As digital technologies continue to transform health communication, understanding how professionals across different national contexts perceive and engage with these tools will be essential for developing effective, contextually appropriate strategies to promote healthy eating across the lifespan.

## Figures and Tables

**Table 1:** Opinions about the importance of selected communication channels for the effective promotion of healthy eating (one-way ANOVAs).

Communication Channels	Total	Poland	France	UK	Germany	F	*p*
Television advertising	3.70	3.84	3.83	3.38	3.38	1.467	0.227
Radio	3.05	2.93	3.17	3.12	3.15	0.406	0.749
Manufacturer’s website	2.81	2.95	2.67	2.85	2.54	0.765	0.516
Retailer website	3.01	3.21	2.72	3.08	2.77	1.649	0.181
Producer profile on social media	3.42	3.70	3.11	3.19	3.54	2.743	0.046 *
Retailer profile on social media	3.36	3.73	2.83	3.27	3.38	**5.132**	**0.002**
Consumer opinions on social media	3.95	4.38	3.64	3.42	4.00	**9.215**	**<0.001**
Nutrition apps for smartphones	3.43	3.73	3.50	2.81	3.15	**6.673**	**<0.001**
Recommendations from family or friends	4.02	4.29	3.72	3.77	4.15	3.318	0.022 *
Dietitian’s recommendations	4.10	4.29	4.11	3.69	4.08	2.965	0.035 *
Dietitian’s website	3.79	4.09	3.75	3.35	3.54	**3.938**	**0.010**
Dietitian’s profile on social media	4.08	4.41	3.86	3.65	4.08	**4.897**	**0.003**
Outdoor advertising (e.g., billboards)	3.20	3.14	3.36	3.31	2.77	1.219	0.306
Shop advertising leaflets	2.75	2.88	2.67	2.62	2.69	0.499	0.684
Articles in the press and magazines	3.28	3.20	3.44	3.46	2.85	1.766	0.157
Cooking programmes on TV	3.34	3.48	3.36	3.27	2.77	1.856	0.140
Cooking blogs	3.18	3.48	3.14	2.92	2.54	**4.491**	**0.005**
Product packaging	3.63	3.86	3.56	3.58	2.92	**3.493**	**0.018**
In-store salesperson recommendations	2.89	3.09	2.81	2.54	3.00	1.839	0.143

Columns 2–6 provide means and their 95% Confidence Intervals. Bold is used to show statistically significant ANOVAs * After applying the Benjamini–Hochberg correction, these ANOVAs no longer meet the significance threshold.

**Table 2:** Topics of campaigns promoting healthy eating among children and young people that should be a priority (%).

Topics	Total	Poland	France	UK	Germany	Chi^2^(3)	*p*
Increasing the consumption of vegetables and fruit	59.5	60.7	55.6	69.2	46.2	2.250	0.522
Increasing the consumption of milk and dairy products	5.3	3.6	0	11.5	15.4	6.944	0.074
Increasing fish consumption	13.7	16.1	5.6	23.1	7.7	4.605	0.203
Increasing fibre intake	23.7	10.7	36.1	42.3	7.7	**15.125**	**0.002**
Increasing dietary diversity	29.0	23.2	36.1	23.1	46.2	4.095	0.251
Limiting the consumption of highly processed foods	60.3	62.5	69.4	50.0	46.2	3.610	0.307
Limiting the consumption of energy drinks	16.8	21.4	11.1	15.4	15.4	1.748	0.626
Limiting sugar consumption	29.0	28.6	27.8	23.1	46.2	2.332	0.506
Limiting salt intake	0.8	1.8	0	0	0	1.350	0.717
Limiting the consumption of saturated fats	2.3	5.4	0	0	0	4.112	0.250
Limiting the consumption of trans fats	4.6	1.8	5.6	7.7	7.7	1.943	0.584
Limiting the consumption of fast food	16.0	28.6	5.6	3.8	15.4	**12.349**	**0.006**
Limiting alcohol consumption	6.1	5.4	8.3	3.8	7.7	0.655	0.884
Limiting the consumption of high-calorie snacks	9.2	5.4	19.4	0	15.4	**8.776**	**0.032**
Reading food labels	19.8	23.2	13.9	26.9	7.7	3.228	0.358
Other	3.8	1.8	5.6	0	15.4	6.696	0.082

Note: the respondents were asked to provide 3 most important ones. Bold is used to show statistically significant differences.

**Table 3:** Topics of campaigns promoting healthy eating among adults that should be a priority (%).

Topics	Total	Poland	France	UK	Germany	Chi^2^(3)	*p*
Increasing the consumption of vegetables and fruit	49.6	41.1	52.8	65.4	46.2	4.428	0.219
Increasing the consumption of milk and dairy products	2.3	3.6	0	3.8	0	1.841	0.606
Increasing fish consumption	16.8	25.0	11.1	15.4	0	6.192	0.103
Increasing fibre intake	29.8	14.3	41.7	50.0	23.1	**14.227**	**0.003**
Increasing dietary diversity	38.2	41.1	38.9	19.2	61.5	7.167	0.067
Limiting the consumption of highly processed foods	47.3	42.9	58.3	53.8	23.1	5.708	0.127
Limiting the consumption of energy drinks	2.3	1.8	0	3.8	7.7	2.884	0.410
Limiting sugar consumption	21.4	17.9	16.7	23.1	46.2	5.682	0.128
Limiting salt intake	6.9	14.3	2.8	0	0	**8.632**	**0.035**
Limiting the consumption of saturated fats	4.6	10.7	0	0	0	**8.421**	**0.038**
Limiting the consumption of trans fats	4.6	5.4	2.8	7.7	0	1.545	0.672
Limiting the consumption of fast food	9.2	5.4	16.7	7.7	7.7	3.512	0.319
Limiting alcohol consumption	27.5	37.5	19.4	19.2	23.1	5.002	0.172
Limiting the consumption of high-calorie snacks	9.2	7.1	2.8	7.7	38.5	**15.517**	**0.001**
Reading food labels	27.5	32.1	27.8	23.1	15.4	1.820	0.611
Other	1.5	0	2.8	0	7.7	4.933	0.177

Note: the respondents were asked to provide 3 most important ones. Bold is used to show statistically significant differences.

**Table 4:** Topics of campaigns promoting healthy eating among elderly people that should be a priority (%).

Topics	Total	Poland	France	UK	Germany	Chi^2^(3)	*p*
Increasing the consumption of vegetables and fruit	42.7	44.6	33.3	50.0	46.2	2.006	0.571
Increasing the consumption of milk and dairy products	27.5	21.4	38.9	15.4	46.2	7.564	0.056
Increasing fish consumption	30.5	32.1	41.7	23.1	7.7	6.051	0.109
Increasing fibre intake	29.0	21.4	27.8	46.2	30.8	5.320	0.150
Increasing dietary diversity	49.6	55.4	47.2	34.6	61.5	3.900	0.272
Limiting the consumption of highly processed foods	17.6	12.5	13.9	38.5	7.7	**10.048**	**0.018**
Limiting the consumption of energy drinks	0.8	0	0	3.8	0	4.070	0.254
Limiting sugar consumption	20.6	17.9	16.7	30.8	23.1	2.290	0.514
Limiting salt intake	14.5	23.2	16.7	0	0	**10.178**	**0.017**
Limiting the consumption of saturated fats	13.0	21.4	2.8	7.7	15.4	7.568	0.056
Limiting the consumption of trans fats	5.3	7.1	2.8	7.7	0	1.844	0.605
Limiting the consumption of fast food	0.8	0	0	3.8	0	4.070	0.254
Limiting alcohol consumption	13.7	14.3	13.9	11.5	15.4	0.151	0.985
Limiting the consumption of high-calorie snacks	3.1	3.6	2.8	3.8	0	0.525	0.913
Reading food labels	16.0	19.6	16.7	11.5	7.7	1.615	0.656
Other	13.0	5.4	19.4	11.5	30.8	**7.904**	**0.048**

Note: the respondents were asked to provide 3 most important ones. Bold is used to show statistically significant differences.

**Table 5:** Having a social media profile as a dietitian (or nutritionist) (%).

Social Media	Total	Poland	France	UK	Germany	Chi^2^(3)	*p*
Facebook	61.1	62.5	75.0	50.0	38.5	7.121	0.068
Twitter (X)	4.6	3.6	0	15.4	0	**9.427**	**0.024**
Instagram	55.0	57.1	58.3	57.7	30.8	3.425	0.331
TikTok	12.2	19.6	5.6	11.5	0	6.191	0.103
YouTube	10.7	10.7	11.1	11.5	7.7	0.149	0.985
LinkedIn	52.7	37.5	69.4	76.9	23.1	**19.935**	**<0.001**
Other	3.8	5.4	2.8	0	7.7	2.031	0.566

Bold is used to show statistically significant differences.

## Data Availability

The original data presented in the study are openly available in Zenodo at https://doi.org/10.5281/zenodo.18141494.

## Appendix A

**Table A1:** Descriptive statistics for opinions about the importance of selected communication channels for the effective promotion of healthy eating.

		Mean	Standard Deviation	95% Confidence Interval for Mean	
				Lower Bound	Upper Bound
Television advertising	Poland	3.84	1.12	3.54	4.14
	France	3.83	1.11	3.46	4.21
	UK	3.38	1.20	2.90	3.87
	Germany	3.38	1.04	2.75	4.02
	Total	3.70	1.13	3.51	3.90
Radio	Poland	2.93	1.14	2.62	3.23
	France	3.17	1.21	2.76	3.58
	UK	3.12	1.07	2.68	3.55
	Germany	3.15	0.99	2.56	3.75
	Total	3.05	1.13	2.86	3.25
Manufacturer’s website	Poland	2.95	1.10	2.65	3.24
	France	2.67	1.04	2.31	3.02
	UK	2.85	1.32	2.31	3.38
	Germany	2.54	0.66	2.14	2.94
	Total	2.81	1.10	2.62	3.00
Retailer website	Poland	3.21	1.11	2.92	3.51
	France	2.72	1.11	2.35	3.10
	UK	3.08	1.32	2.54	3.61
	Germany	2.77	0.60	2.41	3.13
	Total	3.01	1.13	2.81	3.20
Producer profile on social media	Poland	3.70	1.03	3.42	3.97
	France	3.11	0.98	2.78	3.44
	UK	3.19	1.27	2.68	3.70
	Germany	3.54	0.97	2.95	4.12
	Total	3.42	1.08	3.23	3.61
Retailer profile on social media	Poland	3.73	1.00	3.46	4.00
	France	2.83	1.03	2.49	3.18
	UK	3.27	1.31	2.74	3.80
	Germany	3.38	1.04	2.75	4.02
	Total	3.36	1.13	3.16	3.55
Consumer opinions on social media	Poland	4.38	0.70	4.19	4.56
	France	3.64	0.90	3.33	3.94
	UK	3.42	1.06	2.99	3.85
	Germany	4.00	1.00	3.40	4.60
	Total	3.95	0.95	3.78	4.11
Nutrition apps for smartphones	Poland	3.73	0.92	3.48	3.98
	France	3.50	1.00	3.16	3.84
	UK	2.81	0.80	2.48	3.13
	Germany	3.15	0.69	2.74	3.57
	Total	3.43	0.96	3.26	3.59
Recommendations from family or friends	Poland	4.29	0.93	4.04	4.53
	France	3.72	0.94	3.40	4.04
	UK	3.77	0.95	3.39	4.15
	Germany	4.15	1.07	3.51	4.80
	Total	4.02	0.98	3.85	4.18
Dietitian’s recommendations	Poland	4.29	0.73	4.09	4.48
	France	4.11	0.95	3.79	4.43
	UK	3.69	0.88	3.34	4.05
	Germany	4.08	0.86	3.56	4.60
	Total	4.10	0.86	3.95	4.25
Dietitian’s website	Poland	4.09	0.88	3.85	4.32
	France	3.75	1.05	3.39	4.11
	UK	3.35	0.94	2.97	3.72
	Germany	3.54	1.13	2.86	4.22
	Total	3.79	1.00	3.62	3.97
Dietitian’s profile on social media	Poland	4.41	0.85	4.18	4.64
	France	3.86	0.96	3.54	4.19
	UK	3.65	0.98	3.26	4.05
	Germany	4.08	1.04	3.45	4.70
	Total	4.08	0.97	3.91	4.24
Outdoor advertising (e.g., billboards)	Poland	3.14	1.00	2.88	3.41
	France	3.36	0.96	3.04	3.69
	UK	3.31	1.26	2.80	3.82
	Germany	2.77	0.73	2.33	3.21
	Total	3.20	1.03	3.02	3.38
Shop advertising leaflets	Poland	2.88	1.18	2.56	3.19
	France	2.67	0.89	2.36	2.97
	UK	2.62	1.06	2.19	3.04
	Germany	2.69	0.75	2.24	3.15
	Total	2.75	1.04	2.57	2.93
Articles in the press and magazines	Poland	3.20	1.07	2.91	3.48
	France	3.44	0.73	3.20	3.69
	UK	3.46	0.95	3.08	3.84
	Germany	2.85	0.80	2.36	3.33
	Total	3.28	0.95	3.12	3.45
Cooking programs on TV	Poland	3.48	1.03	3.21	3.76
	France	3.36	0.93	3.05	3.68
	UK	3.27	1.04	2.85	3.69
	Germany	2.77	0.93	2.21	3.33
	Total	3.34	1.00	3.16	3.51
Cooking blogs	Poland	3.48	1.03	3.21	3.76
	France	3.14	0.87	2.85	3.43
	UK	2.92	0.93	2.55	3.30
	Germany	2.54	0.88	2.01	3.07
	Total	3.18	0.99	3.01	3.35
Product packaging	Poland	3.86	0.82	3.64	4.08
	France	3.56	1.08	3.19	3.92
	UK	3.58	1.14	3.12	4.04
	Germany	2.92	0.76	2.46	3.38
	Total	3.63	0.99	3.46	3.80
In-store salesperson recommendations	Poland	3.09	1.00	2.82	3.36
	France	2.81	1.01	2.46	3.15
	UK	2.54	1.07	2.11	2.97
	Germany	3.00	1.15	2.30	3.70
	Total	2.89	1.04	2.71	3.07
